# Estimating the Epidemiology and Quantifying the Damages of Parental Separation in Children and Adolescents

**DOI:** 10.3389/fpsyg.2016.01611

**Published:** 2016-10-25

**Authors:** Dolores Seijo, Francisca Fariña, Tania Corras, Mercedes Novo, Ramon Arce

**Affiliations:** ^1^Political Science and Sociology, University of Santiago de CompostelaSantiago de Compostela, Spain; ^2^Departamento de Análise e Intervención PsicoSocioEducativa, University of VigoPontevedra, Spain; ^3^Psychology Forensic Service, University of Santiago de CompostelaSantiago de Compostela, Spain

**Keywords:** parental separation, children of divorce, psychological adjustment, behavioral problems, social relations, self-concept, academic performance

## Abstract

Parental separation is linked to multiple negative outcomes for children in all spheres of life. A field study was designed to estimate the epidemiology and to quantify the outcomes on the wellbeing of children from separated parents. Thus, data on socio-economic status, psychological adjustment, behavioral disorders, social relations, self-concept, and academic achievement were gathered from 346 children and adolescents, 173 separated parents, and 173 parents from intact families in the paediatric catchment area of Galicia (Spain). The results showed that parental separation had a significant negative impact on the children’s and adolescents’ family income (increasing the probability of falling below the poverty line); psychological adjustment (i.e., higher scores in anxiety, depression, hostility, paranoid ideation, and interpersonal alienation); social relations (i.e., less self-control in social relations; higher social withdrawal); self-concept (lower levels of academic, emotional, physical, and family self-concept), and academic achievement (lower academic achievement with higher school dropout rates). Moreover, children from separated families had a higher probability of being exposed to gender violence. Epidemiologically, parental separation is associated to the probability of falling below the poverty line 33.9%; being exposed to gender violence 43.2%; and symptoms such as depression, anxiety, hostility, paranoid ideation interpersonal alienation, and social withdrawal, i.e., 20, 17, 27, 20, 19, and 35.5%, respectively. Inversely, self-control in social relations, and academic, emotional, physical, and family self-concept fell to 16, 32, 27, 22, and 37%, respectively. The interrelationship among these variables and the implications of these results for interventions are discussed.

## Introduction

The 4.2 decline in the crude marriage rate in the EU-28 (marriages per 1,000 inhabitants), has been accompanied by a simultaneous 2.0 increase in the crude divorce rate (EUROSTAT, 2015a), resulting in a 0.48 risk of separation. Thus, the crude divorce rate rose 150% between 1965 and 2011. In absolute terms, the number of marriages in 2011 was around 2,100,000 with about 986,000 divorces, with just over half (∼500,000) being divorces involving children (EUROSTAT, 2015b).

Parental separation is linked to multiple negative outcomes for children in all spheres of life, primarily in psychological adjustment, academic performance, behavioral disorders, self-concept, and social adjustment (Amato and Keith, 1991; Amato, 2001). Two hypothesis may explain these outcomes. The first contends that it is a selection process, i.e., negative outcomes are not due to parental separation, but to other factors such as parental incompetence, parental characteristics (e.g., antisocial personality), or genetic predispositions with the effects of separation being spurious. Alternatively, the other hypothesis suggests a causal relationship, i.e., negative effects are the consequence of parental separation. Although a few studies have lent some support to the selection hypothesis, longitudinal studies, and studies with a design controlling factors not germane to separation have also underscored a causal relationship between parental separation and negative outcomes for children (Capaldi and Patterson, 1991; Averdijk et al., 2012; Lacey et al., 2014). Notwithstanding, a high degree of intersubject variability has been observed, i.e., either negative or positive results were found in a minority, but for the majority they remained unchanged, with a greater prevalence in follow-up studies (pre-separation vs. post-separation) of negative versus positive outcomes (Amato and Anthony, 2014). One explanation for the lack of effects is that many of the problems detected in children following parental separation already existed prior to breakup (Sun, 2001), whereas those who highlight the positive results focus on the degree of conflict, and suggest children living with both parents in situations of intense and chronic parental conflict improve after separation (Hanson, 1999). These results cohabit in studies that view separation as a discrete event (comparing pre- vs. post-separation, with legal separation being the classification criterion), and not as a process whose ultimate aim is legal separation. This would explain much of the injury to children registered “prior to separation” (Arce et al., 2005). Moreover, the size of the negative effects in children from separated and intact families appears to vary through time, i.e., high from the 1950s–1979, falling in the 1980s before rising again in the 1990s (Amato, 2001). Thus, the socio-legal context also influences the results of separation in children. As for the long-term effects of sequelae, two empirically supported alternatives have been proposed in the literature (Hetherington, 2006; Lacey et al., 2014), one proposes the negative outcomes of parental separation on children fade over time (the crisis model); whereas the other suggests that, owing to the persistence of stressors (e.g., reduced quality of parent–child relationships; economic disadvantage, academic performance, and occupational status), negative outcomes pervade over time, becoming chronic or consolidated (chronic stress model), throughout a person’s entire life, and leads to the higher probability of marital breakdown in adult partnerships (Dronkers and Härkönen, 2008). However, these outcomes neither remain static, nor have immediate effects, i.e., delayed expression. Thus, unforeseen circumstances such as remarriage can change the sign of the effects (e.g., increased earnings, rising above the poverty threshold, appearance of new stressors), whereas the appearance of negative outcomes may not be immediate, i.e., deferred expressions (American Psychiatric Association, 2013). In short, though the relation reported in the literature between parental separation and the development of disorders in children was influenced by study design, there is no doubt it entails real and significant negative effects, though these are restricted to only part of the population of children from separated families, whose prevalence and size of injury has as yet to be estimated.

Several theories and models have attempted to explain the results, e.g., the family stress model, family systems theory, life course theory, or social capital theory, which rest on the constructs of stress, coping, risk, and resiliency. Though they all enjoy a degree of plausibility and empirical support, they are nonetheless tenuous. Drawing from these sources, Amato (2000) designed the Divorce-stress-adjustment perspective that views separation as a process that begins before it actually occurs (legal separation), and may continue long after. In order to further explain individual differences in the impact of separation, stressors, and mediators have been introduced (e.g., changing school, a fall in income, continuous exposure to parental conflict, exposure to long-drawn-out parental litigation), which serve to further aggravate injury associated to separation (Ross and Mirowsky, 1999; Lacey et al., 2014); and the moderators (e.g., individual, interpersonal and structural resources, reason of separation, demographic characteristics) used to identify which subjects and under what conditions relationships will occur. Thus, depending on the configuration of the moderating factors for each individual, these may act as protectors (resilience), or as risks (vulnerable), explaining the intersubjects variability in the outcomes of parental separation.

Although, parental separation should take place without any consequences for children (true null hypothesis), that is, no adverse outcomes, the results showed separation had significant negative outcomes for children, with an effect size that is traditionally interpreted as small (Cohen, 1988). Cohen himself has pointed out qualitative interpretations must be contextualized so that a small effect size, in a context where confirmation of the null hypothesis is desirable, is really crucial. Thus, these results must be interpreted taking into account the qualitative categories of serious adverse effects, that is, the undesirable adverse reactions of medication (drugs). Even more so, when the results are mean effects where the negative effects have been weighted either positively or negatively in a minority, but remain unchanged for the majority.

Bearing in mind the literature, a field study was designed to contrast the outcomes, i.e., socio-economic, psychological adjustment, behavioral disorders, social relations, self-concept, and academic achievement domains in a sample of Spanish children from separated families as compared to children from intact families. Succinctly, the consolidated/chronic effects were assessed (>1 year after parental separation), epidemiological increases were estimated, the adverse effects associated to parental separation were quantified, and the minimum and maximum epidemiological ranges of the effects were analyzed to determine variability (moderating factors). Finally, the results were generalized to other samples.

## Materials and Methods

### Participants

The sample consisted of 346 children, 183 girls (52.9%), and 163 boys (47.1%); 173 from separated families and 173 from intact families; age range 6–17 years (*M* = 11.69, *SD* = 3.39). For the group of children from separated families the mean time-lapse since parental separation was 6.72 years (*SD* = 3.90), with a 1-year minimum time-lapse since the legal separation.

### Measures

Parents’ self-reports of annual income were corroborated with their annual tax declarations (joint income or the sum of both independent incomes), to determine the total income of the family unit. Pre- and post-separation data was obtained. The raw data was transformed into categorical variable (income below the poverty threshold or above poverty threshold). The criteria for defining the relative poverty threshold were taken from the Spanish Bureau of Census [Instituto Nacional de Estadística]^1^, and from the Dossiers on poverty in Spain by EAPN [European Anti-Poverty Network, Spain] ^2^.

As for psychological adjustment, the Spanish adaptation (Derogatis, 1977) of the Symptom Check List 90-R (SCL-90-R) was administered to adolescents older than 13 years of age. The checklist consisted of 90 items assessing nine primary symptom dimensions (somatization, α = 0.86, obsessive-compulsive, α = 0.86, interpersonal sensitivity, α = 0.83, depression, α = 0.90, anxiety, α = 0.85, hostility, α = 0.84, phobic anxiety, α = 0.82, paranoid ideation, α = 0.80, psychoticism, α = 0.77), and three global distress indexes (global severity index, positive symptom distress index, and positive symptom total). Participants were required to rate their psychopathological disorders and symptoms on a 5-point Likert-type scale ranging from “not at all” (0), “a little bit” (1), “moderately” (2), “quite a bit” (3) to “extremely” (4).

Socialization was evaluated using the BAS-3 Socialization Battery (Silva and Martorell, 1989), applied to adolescents (minimum 12 years) self-report consisting of 75 items on a yes/no response format. It is structured around five dimensions: consideration for others (α = 0.82, social sensitivity or concern for others); self-control in social relations (α = 0.78, measuring a bipolar dimension representing at one end the positive dimension, i.e., compliance with social rules and norms fostering peaceful coexistence; and at the other the negative dimension, i.e., aggressive, dominant, stubborn, and disobedient); social withdrawal (α = 0.81, active and passive alienation); social/shyness anxiety (α = 0.78, detecting different manifestations of anxiety, fear, and nervousness together with shyness in social relations); and leadership (α = 0.73, ascendency, popularity, initiative, and self-confidence).

Self-concept was evaluated using the Forma 5 [AF-5] Self-concept questionnaire (García and Musitu, 2014), a self-report questionnaire for 12-year-olds and older, consisting of 30 items scored on a 3-point Likert-type scale ranging from “always” (1), “a little bit” (2), to “never” (3). A total of five factors were measured: academic (α = 0.88; self-perception of the quality of their work as a student); social (α = 0.71; social relations); emotional (α = 0.73; emotional states and responses to specific situations, commitment and involvement to some degree in everyday life); physical (α = 0.76; self-perception of their physical aspect and condition); and family (α = 0.80, implication, participation, and integration within the family).

Academic performance was self-reported by children as either good or bad, given that the evaluation scales and levels varied from grade to another. Moreover, self-reports of dropping out of school, in response to the question on whether they had repeated a grade, was crosschecked with the parents, and full consistency was observed. This was only applicable to 8-year-olds and older (under the Spanish education system children under the age of 8 years cannot fail a grade, and from 10 to 12 years, they may repeat a grade only once every 2 years, and from 12 years onward students may repeat a grade on a yearly basis).

Behavioral disorders were measured in two context, i.e., school and social relations, using the disobedience subscales (disruptive behavior in class, α = 0.82), and social aggressiveness (confrontation, arguments, and verbal and physical assaults, α = 0.77) of the TAMAI (Hernández-Guanir, 2015). The norms of the test classify the behavioral disorders as not confirmed (percentile 60 or lower) and confirmed (percentile 61 or higher).

### Procedure and Design

This study was approved by the Clinical Research Ethics Committee of the Autonomous Community of Galicia (Spain). The families and participants were selected from hospital and Primary Healthcare pediatric services in Galicia. During their appointments, parents were explained by the pediatrician the objectives of the study and were asked to collaborate. A member of the research team contacted parents who had freely volunteered to participate in the study, and had signed informed consent. For each separated family with children (remarriage was excluded) a control with similar socio-demographic characteristics [e.g., children’s gender and age prior to separation, economic status, family size, location (urban, suburban, rural), school type (state, private), and parent’s education]. Thereafter, the measurement instruments were administered by rotating the order of administration. No case exceeded 50 min of continuous evaluation. Data were processed in compliance with the Spanish Data Protection Law to guarantee the privacy and non-identification of people or families.

A quasi-experimental research methodology to contrast the consequences of parental separation in children and adolescents was performed. As for design sensitivity analysis, the probability of detecting (1-β) significant differences (α < 0.05) for a small effect size (Amato and Keith, 1991; Amato, 2001) of an association between two variables (*df* = 1, *N* > 180) was >98%; and for *F* test between two groups (numerator *df* = 9; denominator *d* = 145) was >98%.

### Data Analysis

Associations between variables were estimated by the chi-squared for independent or related samples accordingly, and effect size was estimated by the Odds Ratio (OR).

Increases in adverse effects were estimated using to the Binomial Effect Size Display (Rosnow and Rosenthal, 1996), transforming the effect sizes of Cohen’s *d* or OR to *r*, or by obtaining the effect sizes directly from *r*, and the confidence intervals transformed into *r* to *z* (Fisher’s transformation). The increase in adverse effects in samples related to categorical variables was calculated in terms of proportions and their confidence intervals. The size of injury was interpreted in terms of categories of adverse reactions or undesired effects: very frequent/very important (≥1/10), frequent/important (≥1/100 to <1/10), not frequent/not important (≥1/1000 to <1/100), rare/scarce (≥1/10,000 to 1/1000), very rare/very scarce (<1/10,000; World Health Organization, n.d.).

As for mean comparisons, MANOVAs were performed with Pillai**-**Bartlett trace as the multivariate test statistic given that it is more robust to the heterogeneity effects of the variance matrices (Olson, 1976). Though variance homogeneity is not an important requisite when dealing with similar sized groups (big/small <1.5), it was analyzed to determine if it supported or rejected the hypothesis by comparing the theoretical *F* (of the homogeneity test) with the empirical one: if the theoretical *F* is smaller than the empirical one, the alternative hypothesis is substantiated, and vice versa (Palmer, 1996). The effect sizes were estimated as “partial eta-squared” for multivariate contrasts and Cohen’s *d* (Glass delta when heterogeneity of variance was observed: Glass et al., 1981, p. 29), with the confidence intervals, CIs (when 95% CIs do not include zero, the results may be generalized to other samples with a 97.5% probability), derived from the formula of Hedges and Olkin (1985).

## Results

### Socio-Economic Consequences of Parental Separation

The probability of falling below the poverty line is significantly increased by parental separation, χ^2^(1, *N* = 186) = 22.42, *p* < 0.001. The probability of separated families (0.645) of falling below the poverty threshold is twice (OR = 2.11) in contrast to pre-parental separation (0.306). These results are generalizable to other post-separation samples with a probability of 97.5%, 95% CI [0.574, 0.710]. Epidemiologically, this entails an increase in the poverty incidence rate of 33.9% (0.339), 95% CI [0.275, 0.409], ranging with a 95% probability from 27.5 to 40.9%.

### Psychological Adjustment Consequences of Parental Separation

The results showed an effect of parental separation on the psychological adjustment of children (clinical dimensions), Pillai’s Trace = 0.13, *F*(9,143) = 2.31, *p* < 0.05, 1-β = 0.896, explaining 12.7% of the variance of mental health, η_p_^2^ = 0.127. Moreover, parental separation also had effects on global distress, Pillai’s Trace = 0.05, *F*(3,148) = 2.75, *p* < 0.05, 1-β = 0.656, explaining 5.3% of the variance, η_p_^2^ = 0.053.

The univariate effects (**Table 1**) showed that, in comparison to children from intact families, children from separated families exhibited higher levels of depression, anxiety (generalized), hostility (i.e., aggression, anger, fury, irritability, rage, resentment), paranoid ideation (i.e., suspicious, fear of losing autonomy, need of control, difficulties in expressing their hostility), and psychoticism (in non-psychiatric populations it is associated to interpersonal alienation, i.e., feeling different to others, feeling mistreated, misunderstood, unwanted, finding it difficult to express their hostility or in extreme cases the belief that someone is trying physically harm them). Epidemiologically, parental separation was responsible for 20, 17, 27, 20, and 19% increases in symptomatology in depressive, anxiety, hostility, persecutory ideas and interpersonal alienation, respectively. Moreover, parental separation entailed greater global severity distress (GSI), which increased by 17%. Furthermore, the 95% CIs for *r* showed, with a 95% probability, that injury ranged from 4.3 to 38.4% for depression; from 1.2 to 32% for anxiety; from 11.6 to 41.1% for hostility; from 4.3 to 34.8% for paranoid ideation; from 3.2 to 33.8% for psychoticism; and from 1.2 to 32% for global distress. As the mean effect sizes (*d*) for depression, anxiety, hostility, paranoid ideation, psychoticism, and global distress were significantly positive (more symptomatology and clinical severity), and the confidence intervals did not include zero, the results, i.e., significant positive effects of parental separation on children were generalizable to other samples with a probability of 97.5%.

**Table 1:** Univariate effects on the clinical dimensions and distress global indices for the sample factor (separated vs. intact family).

Variable	*F*	*M*_sf_	*M*_if_	*d* (95% CI_d_)	*r* (95% CI_r_)
**Clinical dimensions**					
Somatisation	2.24	0.74	0.59	0.24 (-0.079, 0.559)	0.12 (-0.039, 0.274)
Obsessive-compulsive	0.05	1.12	1.09	0.04 (-0.277, 0.357)	0.02 (-0.139, 0.178)
Interpersonal sensitivity	0.52	1.02	0.94	0.11 (-0.208, 0.428)	0.05 (-0.110, 0.207)
Depression	5.98^∗^	1.07	0.77	0.40 (0.079, 0.721)	0.20 (0.043, 0.348)
Anxiety	4.60^∗^	0.87	0.63	0.35 (0.030, 0.670)	0.17 (0.012, 0.320)
Hostility	12.09^∗∗∗^	1.09	0.62	0.56 (0.236, 0.884)	0.27 (0.116, 0.411)
Phobic anxiety	2.23	0.55	0.41	0.23 (-0.089, 0.549)	0.11 (-0.050, 0.264)
Paranoid ideation	6.32^∗^	1.01	0.73	0.40 (0.079, 0.721)	0.20 (0.043, 0.348)
Psychoticism	5.89^∗^	0.57	0.35	0.39 (0.070, 0.710)	0.19 (0.032, 0.338)
**Distress global indices**					
Global severity index (GSI)	4.42^∗^	0.89	0.69	0.35 (0.030, 0.670)	0.17 (0.012, 0.320)
Positive symptom total (PST)	1.23	38.97	35.37	0.18 (-0.141, 0.501)	0.09 (-0.071, 0.246)
Positive symptom distress index (PSDI)	1.62	2.83	1.66	0.20 (-0.118, 0.518)	0.10 (-0.060, 0.254)

**df*(1, 151); ^∗^*p* < 0.05; ^∗∗∗^*p* < 0.001; *M*_*sf*_, mean of the separated family group; *M*_*if*_, mean of the intact family group.*

### Impact of Parental Separation on Social Relations

The results of a MANOVA showed a significant multivariate effect of the sample factor (separated family vs. intact family) on the socialization of children, Pillai’s Trace = 0.08, *F*(5,146) = 2.36, *p* < 0.05, 1-β = 0.741, with the sample explaining 7.5% of socialization, η_p_^2^ = 0.075.

The univariate effects on the dimensions of socialization showed children from separated families exhibited less self-control in social relation, i.e., less compliant with social rules and norms fostering peaceful coexistence, with an estimated loss of 16%, ranging with a 95% probability from 1 to 31% (**Table 2**). Moreover, the results revealed more social withdrawal in children from separated families as compared to intact families, i.e., they were actively or passively alienated from others with a higher mean than in intact families 21%, ranging with a 95% probability from a minimum of 4.9 to a maximum of 35.5%. As the mean effect sizes (*d*) in self-control and social withdrawal were significantly negative, and the confidence intervals did not include zero, the results, i.e., significant negative effects of parental separation on children in self-control, and social withdrawal were generalizable to other samples with a probability of 97.5%.

**Table 2:** Univariate effects on the socialization for the sample factor (separated vs. intact family).

Variable	*F*	*M*_sf_	*M*_mf_	*d* (95% CI_d_)	*r* (95% CI_r_)
Consideration	0.14	12.48	12.60	-0.05 (-0.369, 0.269)	-0.03 (-0.189, 0.130)
Self-control	4.04^∗^	9.14	10.15	-0.33 (-0.646, -0.014)	-0.16 (-0.310, -0.001)
Social withdrawal	6.83^∗∗^	3.17	2.10	0.42 (0.735, 0.105)	0.21 (0.355, 0.049)
Anxiety-shyness	0.79	3.87	3.51	0.14 (-0.179, 0.459)	0.07 (-0.091, 0.227)
Leadership	1.44	5.09	4.64	0.19 (-0.129, 0.509)	0.09 (-0.071, 0.228)

**df*(1, 150); ^∗^*p* < 0.05; ^∗∗^*p* < 0.01; *M*_*sf*_, mean of the separated family group; *M*_*if*_, mean of the intact family group.*

No effects were observed for the sample factor in consideration, anxiety-shyness and leadership.

### Self-Concept and Impact of Parental Separation

A MANOVA was performed on self-concept with the sample factor (separated family vs. intact family), the results show the sample factor had a significant effect on self-concept, Pillai’s Trace = 0.23, *F*(5,146) = 8.85, *p* < 0.001, 1-β = 1.00, explaining 23.2%, η_p_^2^ = 0.232, of self-concept.

The univariate effects on the dimensions of socialization revealed (**Table 3**) children from separated homes had a low academic self-concept (i.e., poor perception of academic performance); emotional (i.e., low self-perception of emotional adjustment and emotional regulation); physical (i.e., low capacity and poor physical appearance), and family (i.e., less of a feeling of adjustment and importance as a member of the family). Epidemiologically, the mean loss linked to parental separation was in academic self-concept 32%; emotional 27%; physical 22%; and family 37%. Furthermore, the 95% CIs for *r* show, with a 95% probability that the loss ranged from 16.9 to 47.5% for academic self-concept; from 11.5 to 41.2% for emotional self-concept; from 6.3 to 36.7% for physical self-concept; and from 19.4 to 47.7% for family self-concept. As the mean effect sizes of (*d*) were significantly negative in academic, emotional, physical, and family self-concept, and the confidence intervals did not include zero, the results, i.e., significant negative effects of parental separation on children, were generalizable to other samples with a probability of 97.5%.

**Table 3:** Univariate effects on the self-concept for the sample factor (separated vs. intact family).

Variable	*F*	*M*_sf_	*M*_if_	*d* (95% CI_d_)	*r* (95% CI_r_)
Academic	17.40^∗∗∗^	5.25	6.58	-0.67 (-0.979, -0.361)	-0.32 (-0.457, -0.169)
Social	0.32	7.05	7.21	-0.09 (-0.408, 0.228)	-0.05 (-0.208, 0.111)
Emotional	11.91^∗∗∗^	4.96	6.02	-0.56 (-0.872, -0.248)	-0.27 (-0.412, -0.115)
Physical	8.22^∗∗^	5.46	6.35	-0.46 (-0.774, -0.146)	-0.22 (-0.367, -0.063)
Family	24.27^∗∗∗^	7.33	8.70	-0.80 (-1.11, -0.495)	-0.37 (-0.477, -0.194)

**df*(1, 150); ^∗∗^*p* < 0.01; ^∗∗∗^*p* < 0.001; *M*_*sf*_, mean of the separated family group; *M*_*if*_, mean of the intact family group.*

No effects were observed for the sample factor in social self-concept.

### Behavioural Disorders

Parental separation was associated to more disruptive behavior in class (disobedience), χ^2^(1, *N* = 314) = 5.49, *p* < 0.05, φ = 0.132. Briefly, children from separated families more than doubling the probability of disruptive behavior in the class, OR = 2.18, than children from intact families. Moreover, these results were generalizable to other samples with a probability of 97.5%, 95% CI [1.12, 4.24]. Epidemiologically, parental separation increased the mean disruptive behavior in class of 13.2%, 95% IC [0.022, 0.239], with a 95% probability, ranging from 2.2 to 23.9%.

Parental separation was coupled to a significant increase in aggressive behavior in social contexts (social aggressiveness), χ^2^(1, *N* = 320) = 4.47, *p* < 0.05, φ = 0.118. Succinctly, 1.65 more cases of aggressive behavior (OR = 1.65) were reported in children from separated families than intact families. These results were generalizable to other samples with a probability of 97.5%, 95% CI [1.04, 2.64]. Epidemiologically, parental separation was linked to an increase in mean aggressive behavior in social relations of 11.8%, 95% IC [0.009, 0.225], ranging with a 95% probability from 0.9 to 22.5%.

### Impact of Parental Separation on Academic Performance

Self-reported academic performance, measured as either good or bad, was significantly associated to parental separation, χ^2^(1, *N* = 346) = 9.87, *p* < 0.001, φ = 0.169. Thus, children from separated families doubled the probability of negative academic performance, OR = 2.16, than children from intact families. Moreover, these results were generalizable to other samples with a probability of 97.5%, 95% CI [1.33, 3.52]. Epidemiologically, parental separation entailed an increase in the mean incidence rate of academic performance of 16.9%, 95% CI [0.065, 0.270], ranging with a 95% probability from 6.5 to 27%.

School failure, as measured by repeated grades, was associated to parental separation, χ^2^(1, *N* = 181) = 3.85, *p* < 0.05, φ = 0.146, with children from separated families doubling the probabilities of school failure, OR = 2.27, as compared to intact families. Moreover, these results were generalizable to other samples with a probability of 97.5%, 95% CI [0.99, 5.21]. Epidemiologically, parental separation implied an increase in the mean school dropout rate of 14.6%, 95% CI [0.025, 0.263], ranging with a 95% probability from 2.5 to 26.3%.

## Discussion

One of the main distinguishing features of separated families was the significant increase in relative poverty, i.e., insufficient income to cover part or all of the basic needs in Spanish society, which was linked to problems in psychological adjustment (e.g., depression, anxiety), behavioral disorders (e.g., disruptive behavior, deviant behavior, behavioral disorders, aggressive behavior), physical health problems (e.g., obesity), low academic achievers, and a lack of socio-emotional skills (Wadsworth and Achenbach, 2005; Yoshikawa et al., 2012; McLoyd et al., 2014). Parental separation doubled the probability of families falling below the relative poverty line, and the tendency is for this to be prolonged through their entire lives (Lacey et al., 2013), which in turn raises the probability of developing other problems associated to poverty. The size of the increase in mean poverty, and the upper and lower limits were very important (≥1/10).

The negative outcomes of parental separation on the psychological adjustment of children were related to low self-concept (Verrocchio et al., 2015); juvenile and adult behavioral problems (Arce et al., 2010; Novo et al., 2012; Ibabe et al., 2014); lower levels of academic performance (Lacey et al., 2013); material disadvantage (Lacey et al., 2014); more physical health problems (Martinón et al., in press), and the lack of social skills (Arce et al., 2011). Additionally, no differences in social self-concept means that the children assessed their social fit as normal instead of the negative effects of parental separation, linked to deviation risk (Contreras and Cano, 2016). The results showed parental separation led to a mean increase of approximately 20% in depressive symptoms, anxiety (generalized), hostility, paranoid ideation, and interpersonal alienation, as well as a “global severity distress” (GSI), with a very important size of injury (≥1/10), which becomes chronic and is linked to continued exposure to stressors derived from parental separation (Hetherington, 2006). The variability in increased symptomatology was considerable, at the lower limit ranging from 1.2 (anxiety) to 4.3% (depression, paranoid ideation), and an important size of adverse injury (≥1/100 to <1/10), to the upper limit, 38.4% (depression), and an important size of injury, save for hostility that was related to behavioral disorders (Arce et al., 2010), with a lower limit exceeding the important threshold of adverse injury, 11.6%, and the upper limit exceeding it fourfold, 41.1%. In short, parental separation led to a very important injury in psychological adjustment in children in general, being intervention possible and effective (Vázquez et al., 2015).

In terms of the impact on the children’s social skills, parental separation increased social withdrawal, aggressive behavior, dominance, stubbornness, and disobedience (less self-control). This combination undermined facilitating factors (self-control), and intensified inhibitors (social withdrawal) of social competence which in turn lead to deficiencies in problem-solving and conflict management skills, and this to social incompetence (Sestir and Bartholow, 2007; Arce et al., 2010). Mean injury in these social competence factors derived from parental separation was 16% in self-control, and 21% in “social withdrawal,” a very important size of adverse injury, ranging between (lower limit) important adverse effects to (upper limit) effects that tripled the threshold of very important effects (31 and 35.5% for self-control and social withdrawal, respectively). Succinctly, parental separation was linked in general, to los children very important adverse injury in the acquisition of social competency skills.

According to the sign, self-concept acts as either a protective factor or as a risk factor of maladjustment, i.e, in academic performance (Marsh et al., 2014); in emotions, e.g., coping skills (Davis and Humphrey, 2014); in physical capabilities and the assessment of perceived competence (Babic et al., 2014); and the risk of social maladjustment in the family (Arce et al., 2010). The results revealed a very important mean adverse effects for children from separated families in academic (32%), emotional (27%), physical (22%), and family (37%) self-concept. Moreover, the lower limits of adverse effects were very important size in (physical self-concept), and very important in (academic, emotional, and family self-concept); meanwhile the upper limits tripled (physical self-concept), and quadrupled (academic, emotional, and family self-concept) the threshold for very important effects. Briefly, injury in the self-concept of children was very important, i.e., in the region of vulnerability and maladjustment, and affected four dimensions, with no compensatory effect among them (Postigo et al., 2013).

Behavioral disorders correlated with a wide range of risk factors (Guillén et al., 2015; Riglin et al., 2016), that is, they were the consequence of being vulnerable to risk. The results of this study found adverse effects of disruptive and aggressive behavior of a very important mean size in children from separated families. The size of injury ranged from (lower limit) frequent (≥1/100 to <1/10) disruptive behavior at school to not frequent (≥1/1000 to <1/100) aggressive behavior in social contexts, and the (upper limit) twice the very important threshold for effects. Disruptive behavior at school and social aggressiveness were linked to an increasing tendency of social maladjustment (Arce et al., 2010).

Parental separation undermined academic performance and increased school dropout rates, with the mean size of the adverse effects being very frequent (≥1/10), and ranging from important (lower limit) to very important (upper limit). Poor academic performance leads to economic and behavioral problems, relational life course deficits (Arce et al., 2011; Lacey et al., 2014), and subsequently to significant clinical distress or impairment in social, occupational, and other important areas of life (American Psychiatric Association, 2013). Thus, negative outcomes in academic performance are the vehicle to life course negative consequences in other areas.

The results of this study have implications for interventions at three levels: socio-family, school, and personal life. At the socio-family level, parental separation should be planned with the family since parental separation is strongly linked to family poverty (and associated child poverty). At the personal level, children and adolescents from separated families need interventions aimed at preventing and/or repairing injury in social competence resulting from parental separation which puts them at risk of social exclusion, and psychological maladjustment. At school, children from separated families should undergo interventions designed to prevent the negative effects on academic performance and dropout rates. A meta-analytical review (Durlak et al., 2011) of the universal school-based intervention programs to promote students’ social and emotional learning has highlighted that interventions were effective in socio-emotional skills (Hedge’s *g* = 0.57), behavioral disorders (g = -0.22), emotional distress (*g* = -0.24), positive social behavior (*g* = 0.24), and improvement in academic performance (*g* = 0.27). In terms of the efficacy of an intervention, this implied an associated improvement of 10.9, 11.9, 13.4, and 27.4% in socio-emotional skills, behavioral disorders; emotional distress, positive social behavior, and improvement in academic performance, respectively. As problems and deficits occurred both at the personal level (negatives outcomes for parents and children), and the socio-family level (e.g., deficient financial resources, parent-child interaction, and in academic and occupational settings), a multilevel perspective should be adopted to enhance the efficacy of the intervention (individuals – parents, children, new members of the family such as new partners – new families, new social life), and a multimodal cognitive and behavioral approach (Arce and Fariña, 1996).

As for explanatory models that integrate the results, the divorce-stress-adjustment perspective can be complemented by two contrasted models explaining social maladjustment: the additive/accumulative deficits model (Farrington, 1992; Lösel and Bender, 2003), and the “natural developmental trajectory model” (Arce et al., 2010). The accumulative deficits model underscores the adverse outcomes were not restricted to one sole domain, but were combined in an interrelated set. Moreover, the natural developmental trajectory model offers an explanation on the natural course of adverse effects on children due to parental conflict, followed by the initial effects in personal areas (e.g., psychological adjustment, relationships with parents), followed by academic areas that facilitate long-term chronification of adverse effects.

Though the adverse effects found in this study were generalizable to other samples with a high probability (>0.975), and the power of the design was high (>0.98), these may reflect potential cultural differences, particularly in the size and ranges of the adverse effects observed (American Psychiatric Association, 2013), and the size of the adverse effects may vary through time (Amato, 2001). Nevertheless, regardless of the effect sizes, the adverse effects of parental separation on children are significant and extemporal in western cultures (Amato and Keith, 1991; Amato, 2001). The results of this study on poverty were limited to assessing parental income, but the effects derived from additional expenditure were not included (e.g., running two households, legal fees), and often lead to further financial hardship.

Research is required to examine and define the mediating effects of variables such as gender in children and the development of associated consequences (Hetherington et al., 1985); the age of children at the time of separation; the degree of conflict in the separation; and to contrast the pre and post effects following parental separation (Evans et al., 2008; McCloskey and Eisler, 2008).

## Author Contributions

All authors listed, have made substantial, direct and intellectual contribution to the work, and approved it for publication.

## Conflict of Interest Statement

The authors declare that the research was conducted in the absence of any commercial or financial relationships that could be construed as a potential conflict of interest.

## Footnotes

1 http://www.ine.es/

2 www.eapn.es/
